# Drug Resistance Evolution in HIV in the Late 1990s: Hard Sweeps, Soft Sweeps, Clonal Interference and the Accumulation of Drug Resistance Mutations

**DOI:** 10.1534/g3.119.400772

**Published:** 2020-02-19

**Authors:** Kadie-Ann Williams, Pleuni Pennings

**Affiliations:** Department of Biology, San Francisco State University, California, 94132

**Keywords:** clonal interference, drug resistance, evolution, hard sweep, HIV, soft sweep

## Abstract

The evolution of drug resistance in pathogens such as HIV is an important and widely known example in the field of evolutionary medicine. Here, we focus on a unique data set from the late 1990s with multiple viral sequences from multiple time points in 118 patients. We study patterns of evolutionary dynamics in the viral populations in these patients who were treated with Reverse Transcriptase Inhibitors and Protease Inhibitors in the late 1990s. Specifically, we aim to visualize and analyze examples of population genetic processes such as selective sweeps and clonal interference. The figures and descriptions in this paper can be used in evolution and population genetics classes. We show and analyze a wide variety of patterns, specifically: soft sweeps, hard sweeps, softening sweeps and hardening sweeps, simultaneous sweeps, accumulation of mutations and clonal interference.

The evolution of drug resistance in HIV has been studied extensively by clinical researchers and evolutionary biologists alike (Clutter *et al.* 2016; Pennings 2013; Reiss *et al.* 1988; Wensing *et al.* 2015). Research on drug resistance in HIV started in the 1980s when the first available HIV drug (AZT) turned out to be extremely vulnerable to drug resistance evolution (Mayers *et al.* 1992; Reiss *et al.* 1988) Many changes in HIV treatments in the two decades between the development of AZT (Fischl *et al.* 1987) and the development of single-pill combination treatments in 2006 (De Clercq 2006) were aimed at slowing down the evolution of drug resistance. Drug resistance evolution on modern combination therapies has become quite uncommon. For example, a large study that compared the ”quad” pill (based on an integrase inhibitor) with Atripla (based on a non-nucleoside RT inhibitor) found that in both arms of the trial, drug resistance developed in only 2% of patients in the first year of treatment (Sax *et al.* 2012). In evolutionary biology, HIV drug resistance is often used as a key example for the importance of evolutionary processes in textbooks and articles. With the increasing interest in evolutionary medicine (Nesse *et al.* 2009) it is likely that more and more students in science and medicine will learn about evolutionary processes through examples from HIV.

Drug resistance in HIV provides great examples of evolutionary phenomena such as selective sweeps (Messer and Neher 2012; Pennings *et al.* 2014), standing genetic variation (Li *et al.* 2011; Paredes *et al.* 2010; Pennings 2012) and mutation-selection balance (Zanini *et al.* 2017; Theys *et al.* 2018). Even though HIV has been the subject of intensive research and studied by evolutionary biologists for decades, we are still often limited by the availability of high-quality data. Specifically, datasets consisting of multiple sequences at multiple time points are uncommon for HIV. Notable exceptions are the datasets on virus populations in untreated patients described by Shankarappa *et al.* (1999), Zanini *et al.* (2015) and Strauli *et al.* (2019). Another exception, and the only one that focuses on treated patients, is the dataset from Bacheler *et al.* (2000), which we use in this paper. We have used this dataset for two previous studies (Pennings *et al.* 2014; Theys *et al.* 2018). In this manuscript, we show the many different patterns of drug resistance evolution observed in the Bacheler dataset. We will give an overview of the main patterns that can be observed in the evolutionary dynamics in HIV populations in patients on multi-drug treatments. These examples can be used to illustrate important evolutionary processes, such as selective sweeps and clonal interference, that have previously been illustrated by examples from computer simulations or using more indirect evidence. Note that this article is meant to illustrate and describe, not to quantify. We focus on showing the best examples of several phenomena, rather than, for example, trying to determine exactly how often a certain phenomenon occurs.

## Methods

We used sequences from a dataset collected by Bacheler *et al.* (2000), a study that focused on patients in three clinical trials of different treatments, all based on Efavirenz (a non-nucleoside reverse transcriptase inhibitor, NNRTI) in combination with nucleoside reverse transcriptase inhibitors (NRTI) and/or protease inhibitors (PI). Some patients in these trials were initially prescribed monotherapy, which almost always lead to drug resistance, and some patients had previously been treated with some of the drugs, so their viruses were already resistant to some components of the treatment. Viral loads in these patients were typically not suppressed, which made it possible to sequence samples even during therapy. All samples were taken from blood plasma. The authors of the original study used a cloning approach so that “each sequence reflects the genotype of an independent viral genome” (Bacheler *et al.* 2000). Sequencing was done using the conventional (Sanger) method which has a low error rate. From the viral genome the Protease gene and the first 229 codons of Reverse Transcriptase were sequenced. We have previously used part of this dataset to study soft and hard selective sweeps (Pennings *et al.* 2014) and to study fitness costs of transition mutations (Theys *et al.* 2018).

The original dataset consisted of sequences for 172 patients. However, because we are interested in population genetic dynamics over time, we focused on patients with at least two sequences for at least two different sampling dates, and at least 5 sequences in total. This left us with data for 118 patients with a median of 24 sequences and 4.5 timepoints per patient. Sequences were 984 nucleotides long and were composed of the 297 nucleotides that encode the HIV protease protein and the 687 that encode the beginning of RT. Sequences were retrieved from Genbank under accession numbers AY000001 to AY003708.

Drug resistance mutations were used according to the HIV Drug Resistance Database (https://hivdb.stanford.edu/). For each patient, we visualized all observed resistance-related mutations and the codon they are part of. In addition, for the more extensive visualizations, we showed all sites that were polymorphic – as long as a variant was observed at least twice (*i.e.*, we excluded singleton mutations from the visualizations). We then inspected all of the visualizations to determine whether there were convincing examples of different types of sweeps, accumulation resistance mutations or clonal interference. We considered evidence of clonal interference to be when at least one resistance mutation first goes up in frequency and then down in frequency, while at the same time another resistance mutation that is present on a different genetic background (in linkage disequilibrium) increases in frequency and reaches (near) fixation in the population. For the patients in which we found evidence for clonal interference we also created Muller plots (Muller 1932) for the sites involved in clonal interference. All analysis was done in R (R Development Core Team (2011) and code is available on github https://github.com/PenningsLab/ClonalInterferenceHIV.git.

### Data availability

All sequences and R scripts and Supplemental material available at figshare: https://figshare.com/projects/Williams_Pennings_2020_G3_Sweeps_and_Clonal_Interference_HIV/75525.

### Hard selective sweeps

**Figure 1:**
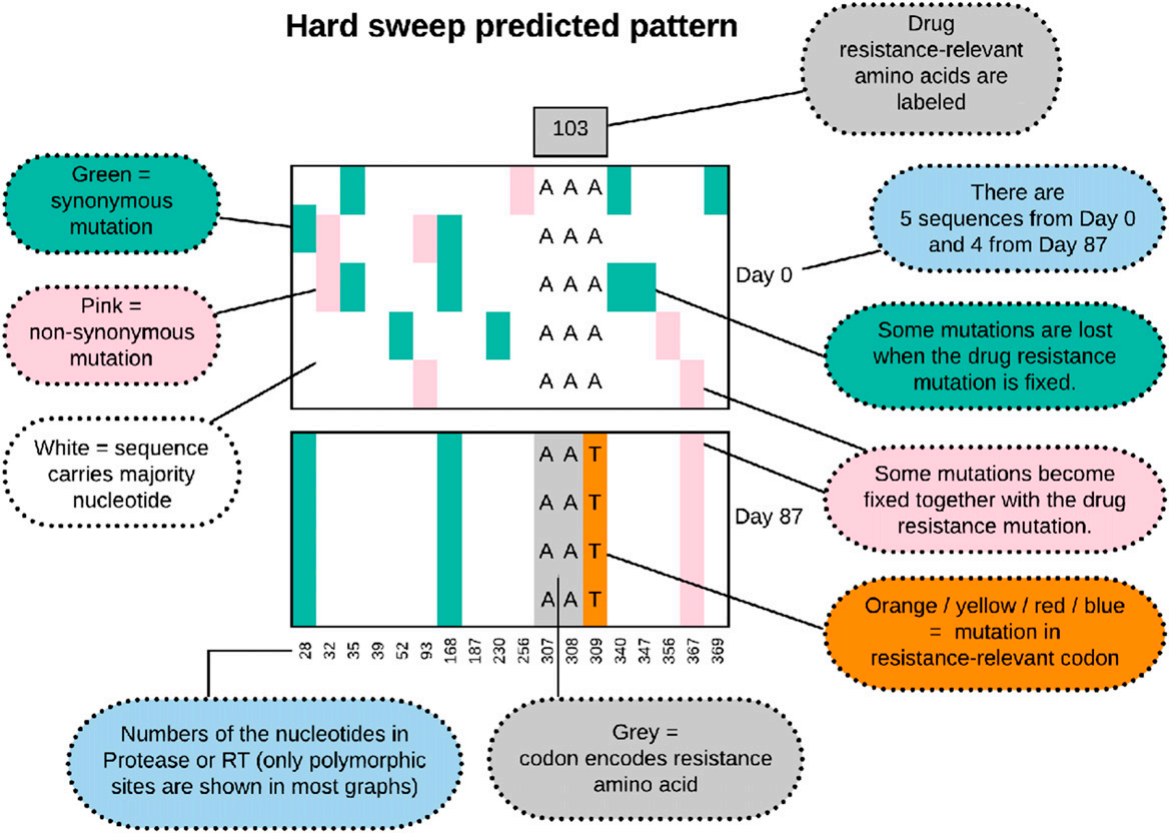
Predicted pattern of a hard sweep, when a single mutant increases in frequency rapidly because it provides a fitness benefit to the virus.

In molecular evolutionary biology, a hard selective sweep is the pattern that results when a single new mutation occurs in the population (usually *de novo*) and fixes in the population due to strong selective pressure Smith and Haigh (1974); Berry *et al.* (1991); Hermisson and Pennings (2017). We show in Figure 1 what we expect to observe in our data when a hard sweep occurs in the viral population within a patient. Note that in all of the figures that show alignments of viral sequences, we show only a subset of the sites – in the early figures we show all polymorphic sites, from Figure 8 onwards we show only the polymorphic drug-resistance related sites. During a hard sweep, we expect to see a drug resistance mutation (here K103N AAT in orange) fix in the population. This mutation changes the AAA codon to the AAT codon, which results in an amino acid change from Lysine to Asparagine, which confers resistance to NNRTI drugs. Second, we expect to observe that over time, some existing mutations (in green and pink) are lost (*e.g.*, position 347) and some are fixed (*e.g.*, position 367) along with the resistance mutation, leading to a much reduced genetic diversity in the sample from day 87 when compared to the sample from day 0. Recombination between the resistance site and other sites, or new mutations that occur after the sweep can make that not all mutations are entirely lost or fixed.

**Figure 2:**
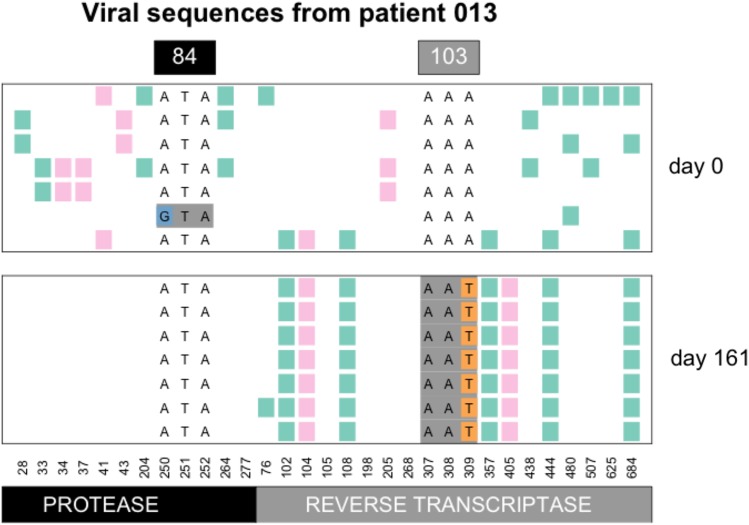
Hard sweep pattern in patient 13. The AAT codon of the K103N mutation, which confers resistance to NNRTI drugs fixes between day 0 and day 161.

In the example of patient 13, we see that the K103N mutation in reverse transcriptase (RT) fixes in the population some time after day 0 (beginning of multi-drug therapy) and before day 161 (see Figure 2). This A→T mutation at the third position of codon 103 (shown in orange) leads to an amino acid change (K103N) which is known to confer drug resistance to NNRTI drugs (Bacheler *et al.* 2000). Here, in patient 13, five synonymous (green) and two non-synonymous (pink) mutations fix along with the resistance mutation, whereas several synonymous and non-synonymous mutations that were seen at day 0 are no longer seen at day 161. The result is a strongly reduced amount of genetic variation at day 161 when compared to day 0. This pattern is known as a classic or hard selective sweep, as predicted by Smith and Haigh (1974). We have previously described and analyzed selective sweeps involving K103N in the Bacheler data set (Pennings *et al.* 2014).

Seeing the pattern of a hard sweep in HIV may be surprising to some because HIV is often seen as a pathogen with high mutation rates and large population sizes (*e.g.*, Boltz *et al.* (2012); Coffin (1995)), which should favor soft sweeps (Hermisson and Pennings 2017). There is indeed a possibility that the sweep in patient 13 was initially a soft sweep and later “hardened” – we have evidence that this can happen and we will discuss hardening of sweeps in more detail later in the manuscript. If we take the observation of a hard sweeps at face value, it suggests that the influx of mutations in some patient viral populations is not as high as one may expect. This could also be due to the treatment working well and reducing the amount of viral replication that can happen (Feder *et al.* 2016). Also note that this selective sweep pattern (with linked mutations up to 180 nucleotides away fixing alongside the resistance mutation) shows that the recombination rate is not high enough to effectively unlink the resistance site from its surroundings during the selective sweep.

**Figure 3:**
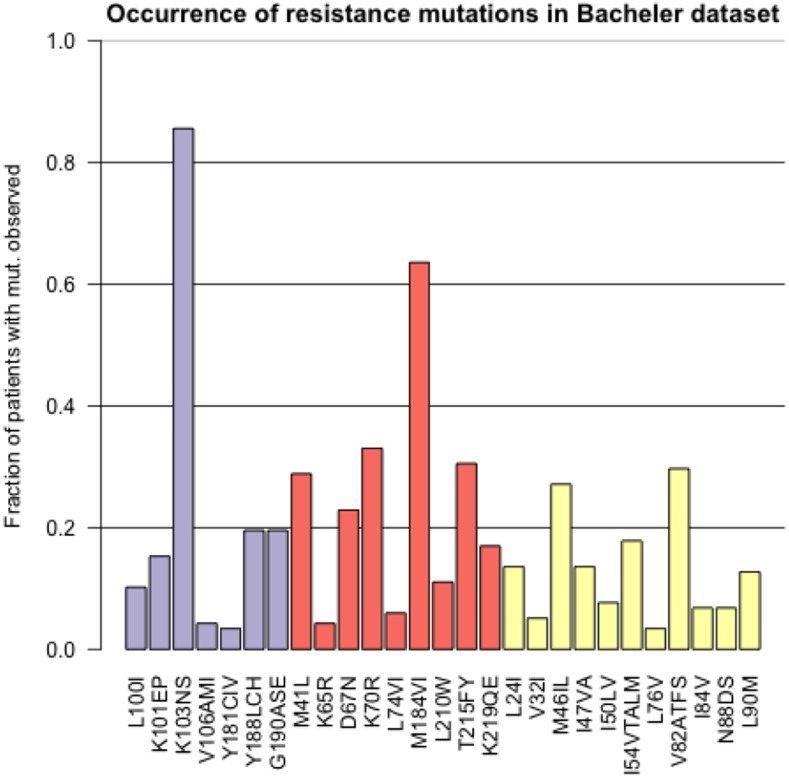
Prevalence of different resistance mutations in the Bacheler dataset with 118 patients. The purple bars are mutations that lead to NNRTI resistance, the red bars are mutations that lead to NRTI resistance and the yellow bars are mutations that lead to PI resistance.

The most common resistance mutation in the Bacheler data set is K103N in reverse transcriptase. This mutation makes the virus highly resistant to the drugs Efavirenz and Nevirapine (according to the HIV Drug Resistance Database, this mutation “reduces Nevirapine and Efavirenz susceptibility by about 50 and 20-fold, respectively.” (Shafer 2009). In Figure 3 we show for the most common resistance mutations, in how many patients they were observed in the dataset. K103N is observed in more than 80% of the patients in the dataset.

**Figure 4:**
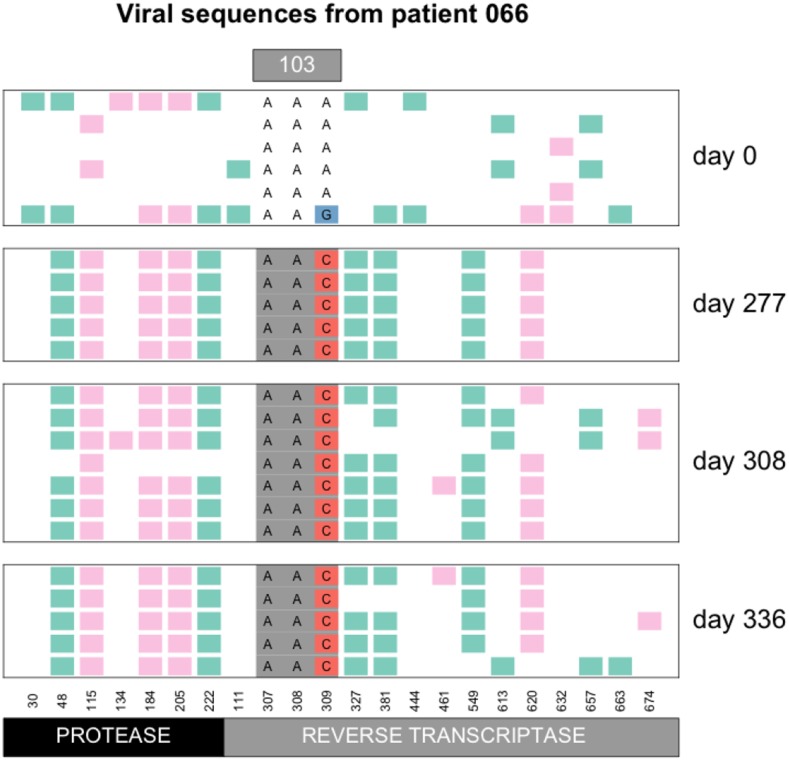
Hard sweep pattern in patient 66. The AAC codon of the K103N mutation, which confers resistance to NNRTI drugs, fixes between day 0 and day 277.

There are two mutations that can create the K103N amino acid change: AAA→AAT and AAA→AAC. In patient 13 the AAT codon fixed (Figure 2), whereas in patient 66, the AAC codon fixed (Figure 4). Overall, we observed the AAC allele more often than the AAT allele. In 56 patients, we observed only the AAC allele, while in 4 patients we observed only the AAT allele, additionally, in 41 patients we observed both alleles (see also next section on soft sweeps from recurrent mutation) and in 17 patients, neither of the two resistance alleles are observed. It is unclear why the AAC allele is more common than the AAT allele, but this has been observed previously (Clutter *et al.* 2016; Palmer *et al.* 2006). The difference in mutation rates from A→C and from A→T is minimal and cannot explain the difference in how often these mutations are observed in the viral sequences (Abram *et al.* 2010; Zanini *et al.* 2017). We have some evidence that the AAT allele is associated with an additional fitness cost (Tenorio, Fryer, Pennings, unpublished results).

### Sweeps at positions other than RT103

Even though K103N is the most common drug resistance mutation in the dataset, there are many other mutations that sweep and leave the classical sweep pattern of reduced variation. For example, in patient 21, mutation Y188L is not observed at day 0, but then it is seen in two of three sequences at day 55 and it is fixed at day 83 (Figure 1, Additional Examples Supplement). The difference in genetic variation between day 0 and day 83 is striking. Note that Y188L requires two mutations (T→C at position 1 and A→T at position 2 of the codon).

### Soft sweeps from recurrent mutation

**Figure 5:**
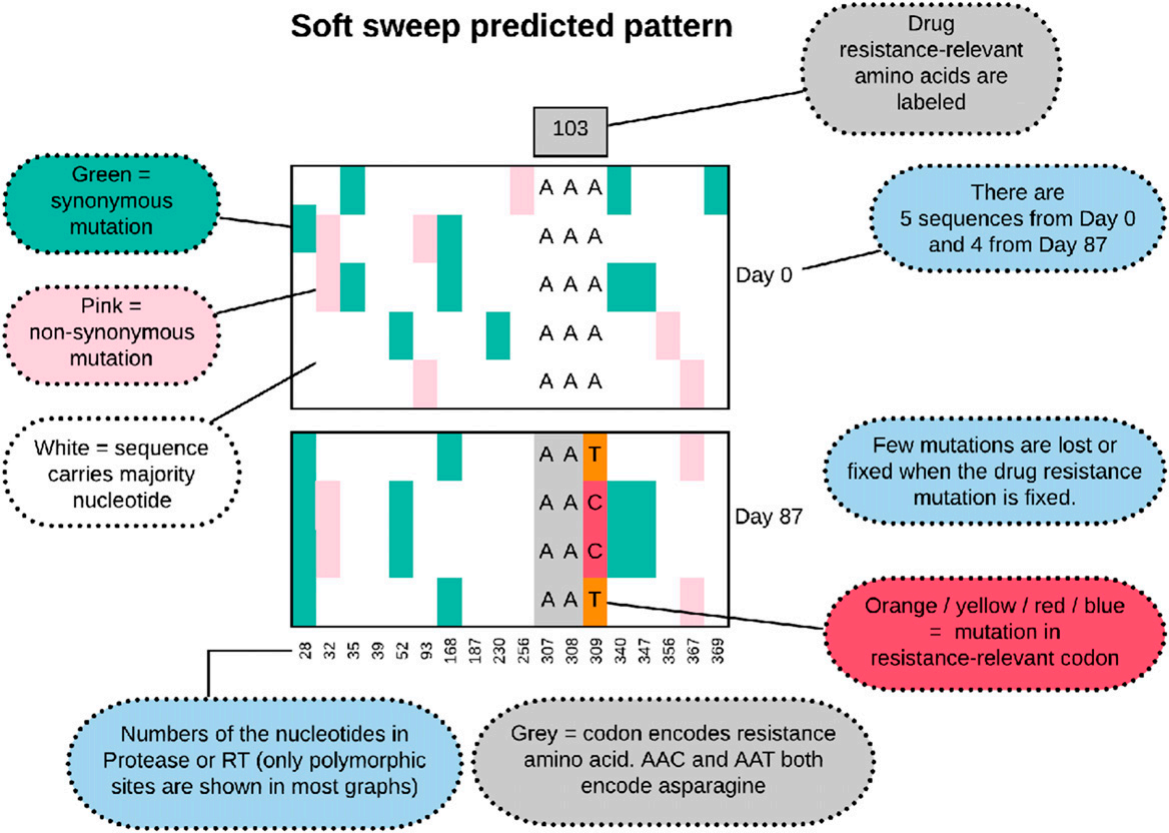
Predicted pattern of a soft sweep from recurrent mutation, when two or more mutants with the same functional effect (here the A→C and A→T at position 309) increase in frequency rapidly.

A soft sweep from recurrent mutation, also known as a multiple-origin soft sweep, occurs when a beneficial mutation occurs more than once in a population and both origins are then observed when a sample is taken when adaptation is (near) complete and the WT is (almost) lost in the population. We expect such soft sweeps to occur in situations where the mutation rate multiplied with the population size is substantial (Hermisson and Pennings 2017). We show in Figure 5 the predicted pattern of what a soft sweep would look like in our data.

**Figure 6:**
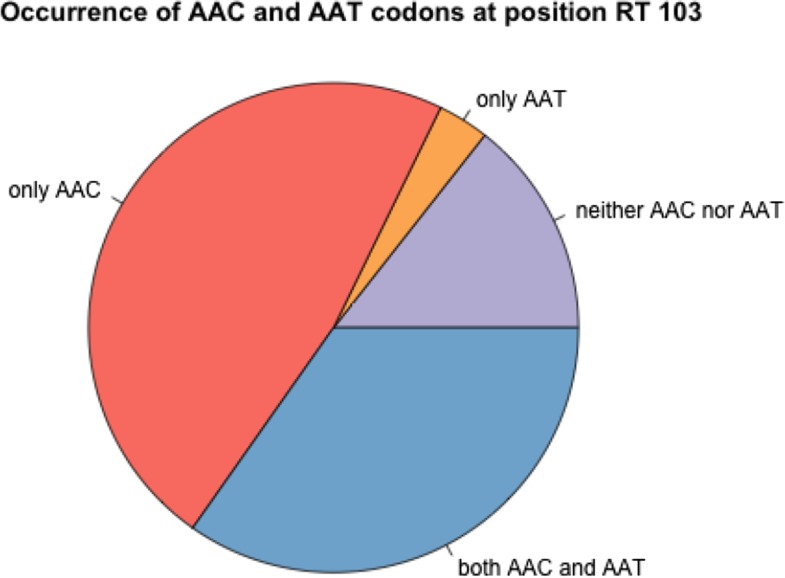
Occurrence of AAC and AAT codons at K103N in 118 patients. In 14% of the patients, we see neither AAC nor AAT, but only the WT codon (AAA) is observed. In 47% of the patients, we observe the resistance codon AAC, in 3% of the patients we see the resistance codon AAC and in 35% of the patients, we see both resistance codons. In all cases we may also see the WT codon, especially in the early time points.

**Figure 7:**
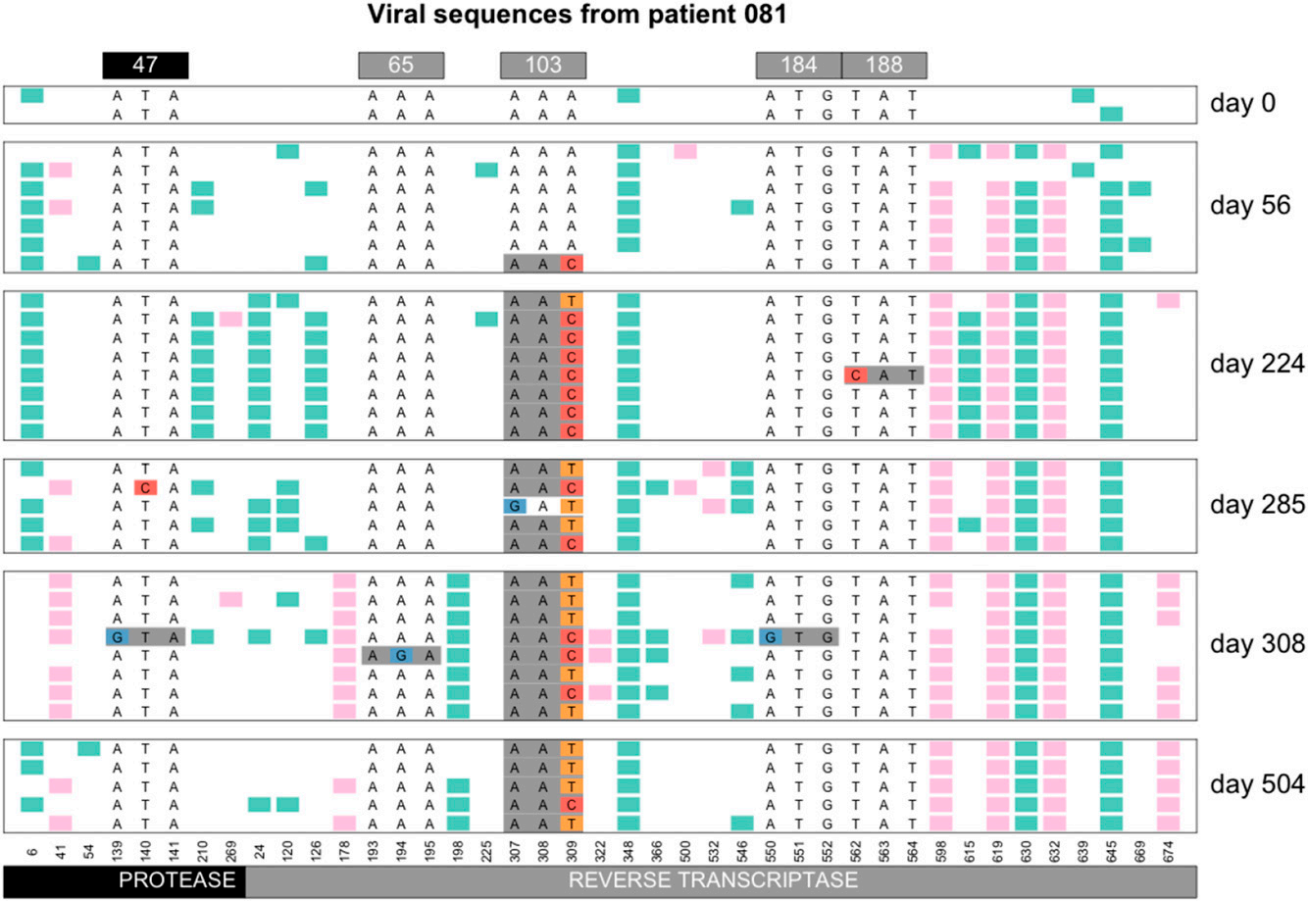
Soft sweep from recurrent mutation in patient 81. At codon 103, both the AAC and the AAT resistance codon are observed.

The commonly observed K103N mutation in reverse transcriptase can be created by an A→T mutation or an A→C mutation. Both of these mutations are tranversion mutations and therefore have a fairly low mutation rate for HIV (mutation rate $5\text{*}10^{-}7$ for A→T and $9\text{*}10^{-}7$ for A→C according to Abram *et al.* (2010)) . In earlier work, we have shown that both soft and hard sweeps occur at this position in the Bacheler dataset (Pennings *et al.* 2014). Hard sweeps were shown in patient 13 (Figure 2) and 66 (Figure 4). However, in one third of the patients (41 out of 118 patients, 35%) both alleles were observed, see Figure 6. When both codons (AAT and AAC) are present at position 103, this provides a clear example of a soft sweep from recurrent mutation (Pennings *et al.* 2014). We see this pattern in patient 81 (Figure 7).

#### “Hardening” of soft sweeps:

**Figure 8:**
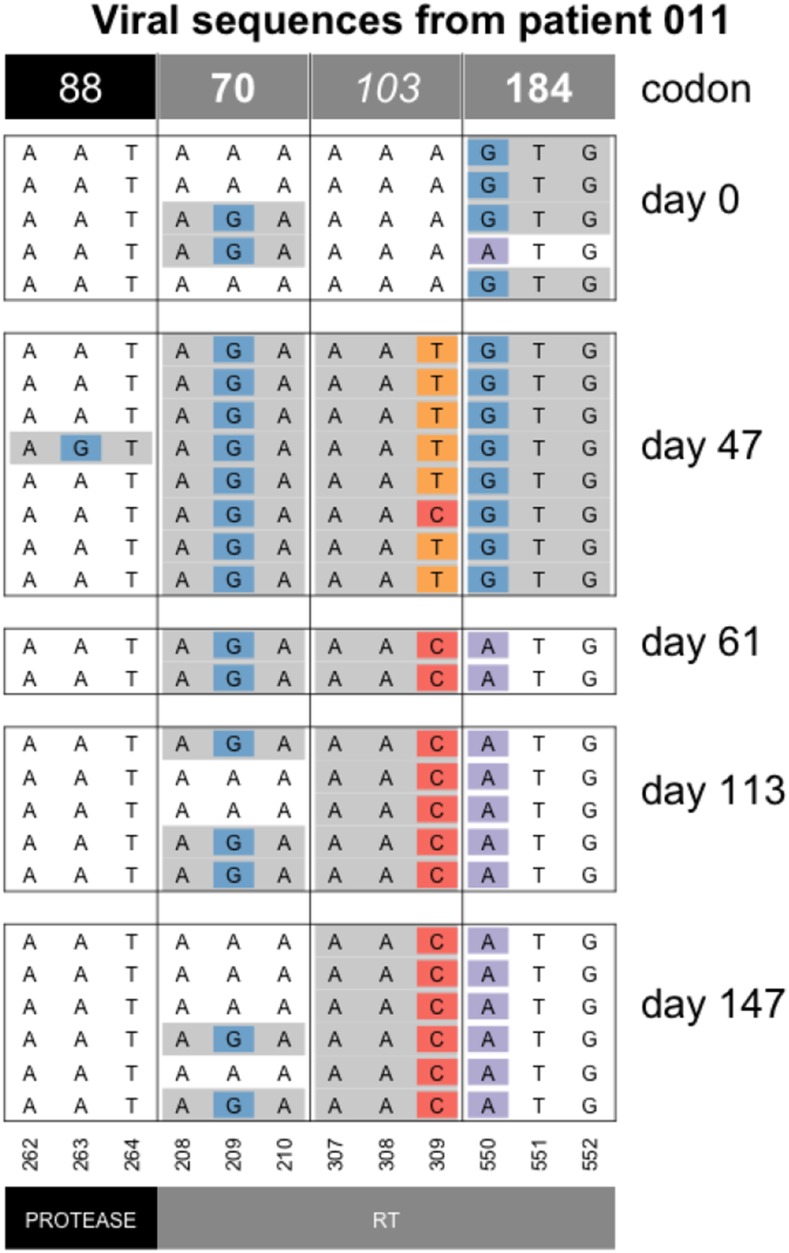
Hardening of soft sweep at K103 in patient 11. At day 47, both the AAT and the AAC codon are present at the 103^rd^ codon of RT, but from day 61 onwards, only the AAC codon is observed. Note that only drug-resistance related codons are shown in this figure.

In 2014, Wilson and colleagues described a scenario where sweeps can start off being soft (due to recurrent mutation) but then become hard due to population bottlenecks. This phenomenon was named the “hardening” of soft sweeps (Wilson *et al.* 2014). In several patients, we observe patterns that are consistent with a hardening of soft sweeps. Here we show an example of the hardening of soft sweeps from recurrent mutation in patient 11 (Figure 8). In this patient, both resistance codons at position 103 are present initially, but one of the two alleles then vanishes (the T), as the other fixes (C). Note that diversity at codon 70 is present at day 113 and 147. This may either be because of a mutation that occurred after the hardening occurred or because of recombination between codons 70 and 103.

The hardening of a sweep where we initially see two selected codons, but later only one remains, is fairly common in the dataset. E.g., we also see it in patients 5, 24, 55, 57, 87 and 170 at position RT103 and in patients 94 and 133 for position RT41 (see Figure 11 for patient 24, the Additional Examples Supplement for patient 24 and supplementary figures for all others). The hardening may be due to demographic bottlenecks, as described in the original paper by Wilson *et al.* but it could also be due to hitchhiking when a new beneficial mutation occurs. For example, in patient 11 (Figure 8), the G→A reversal mutation in RT184 (recreating the WT at that position) may be a beneficial mutation that spreads, such that the AAC allele at RT103 hitchhikes to fixation (Figure 8). The change in selection pressure at the RT184 position is likely due to a change in the treatment for this patient. M184V is a common, but costly mutation, which often appears when a patient is taking the NRTI drug 3TC, but usually disappears within a few months when the patient is no longer on 3TC (Jain *et al.* 2011).

#### “Softening” of hard sweeps:

**Figure 9:**
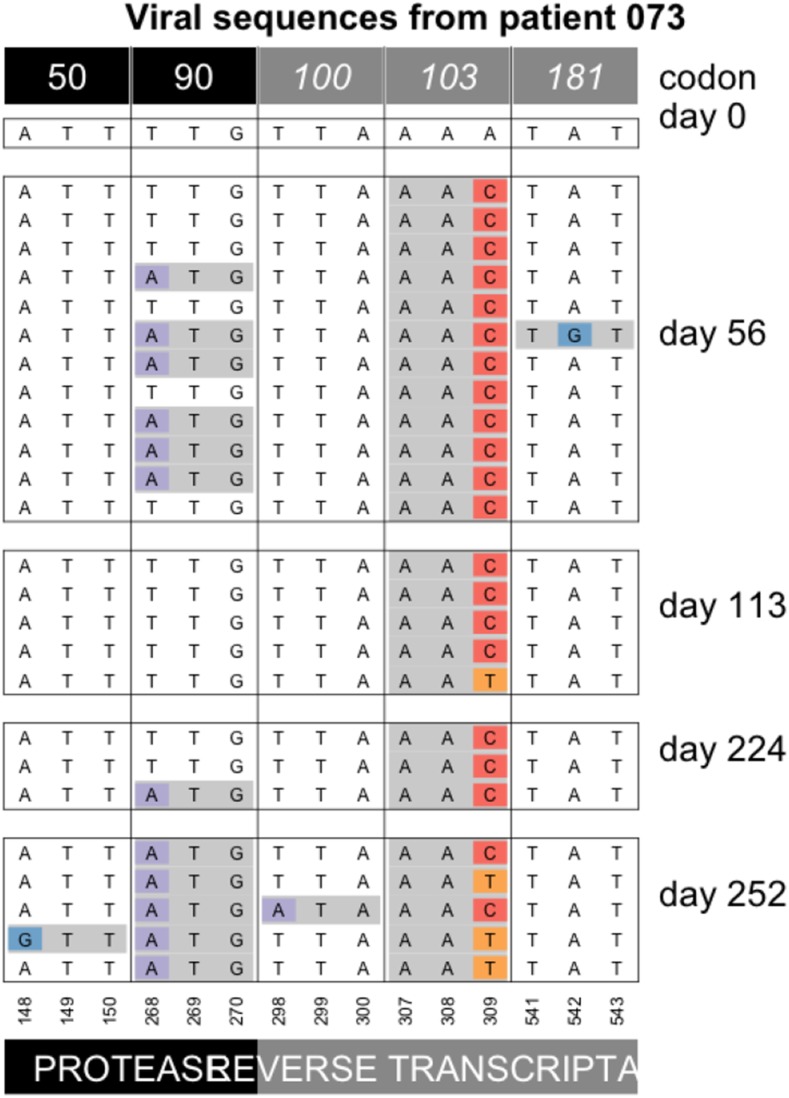
Softening of a hard sweep at K103N in patient 73. Note that only drug-resistance related codons are shown in this figure.

We also see several cases of “softening” of hard sweeps. In these cases, a single mutation is initially seen to reach a high frequency or even fixed in the population, but the alternative allele appears in later samples. In patient 73 the AAC codon at the 103^rd^ codon of RT appears to be fixed in the population at day 56. Later, at days 113 and 252, the alternative codon (AAT) is observed as well (Figure 9). Since AAT is synonymous to the AAC codon, the appearance of the AAT codon is not due to selection for increased drug resistance.

As far as we are aware, this phenomenon of the “softening” of a hard sweep has not been observed before, but Feder *et al.* 2019, predict that this pattern can occur when selection and migration are both strong and sampling is done from a single compartment (deme or subpopulation). What happens is that different beneficial mutations can occur and reach high frequencies in different compartments of the human body such as the brain or the gut, but soon afterward, migration mixes genetic material from the different compartments, so that other beneficial mutations appear in the sampled compartment. In patient 73, the AAC allele is seen fixed on day 56, presumably because the allele fixed in the blood compartment, which is where the samples in this study are from. From day 113, however, the other allele (AAT) is also present. Presumably, this happened because the AAT allele fixed in one or several other compartments, and the AAT alleles that are observed from day 113 are migrants from one or more other, unsampled, compartments. The phenomenon of “softening” of hard sweeps is seen three times in the current dataset. Twice, we see the AAC allele first (patient 73 (Figure 9) and 70 (Figure 5 in Additional Examples Supplement), and one time we see the AAT allele first (patient 93, see Figure 6 in Additional Examples Supplement).

### Sweeps at multiple sites

#### Two mutations increase in frequency simultaneously:

**Figure 10:**
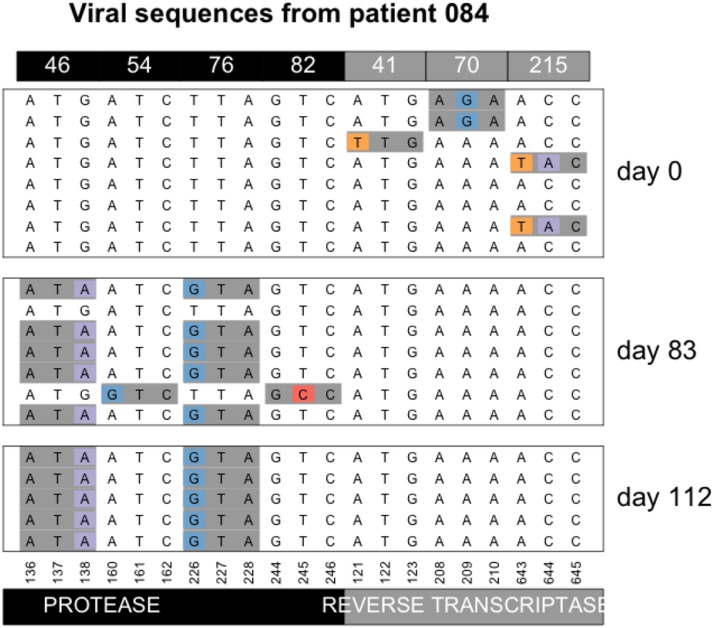
Simultaneous sweep of M46I and L76V in protease in patient 84. Note that only drug-resistance related codons are shown in this figure.

In a previous paper that was also based on data from the Bacheler dataset, we argued that most sweeps involve a single beneficial mutation (Pennings *et al.* 2014). It should be noted that even if this is strictly true, sampling may not be done often enough to observe each individual sweep. For example, in patient 70 (Figure 5 Additional Examples Supplement), resistance mutations at protease codons 54 and 82 are fixed in the population on day 169, even though they were absent in the previous sample (day 56). The data cannot tell us whether these two mutations fixed at the same time or one after the other. However, in the current dataset, we also have two examples of mutations in different codons that can be observed to increase in frequency simultaneously. In both cases, we see the simultaneous increase in frequency before the mutations reach fixation. In patient 84, mutations M46I and L76V in protease are not seen at day 0 (Figure 10), but on day 83, they both appear on 5 out of 7 sequences (71% frequency) in perfect linkage disequilibrium. Later, on day 112, they are both fixed.

The second example of a simultaneous sweep is seen in patient 100 (Figure 7 Additional Examples Supplement). Mutations D67N and K70R appear to be sweeping simultaneously in perfect linkage disequilibrium, though in this case we don’t observe the start of the sweep.

These two examples of simultaneous sweeps are important because they show that it is possible for multiple mutations to sweep simultaneously even if this is not the most common mode of adaptation. Note that in both cases, the two mutations involved confer resistance to one class of drugs (two PI mutations in the first example and two NRTI mutations in the second example), and they are mutations that are often seen to occur together, presumably because of an epistatic interaction between them.

#### Accumulation of resistance mutations:

**Figure 11:**
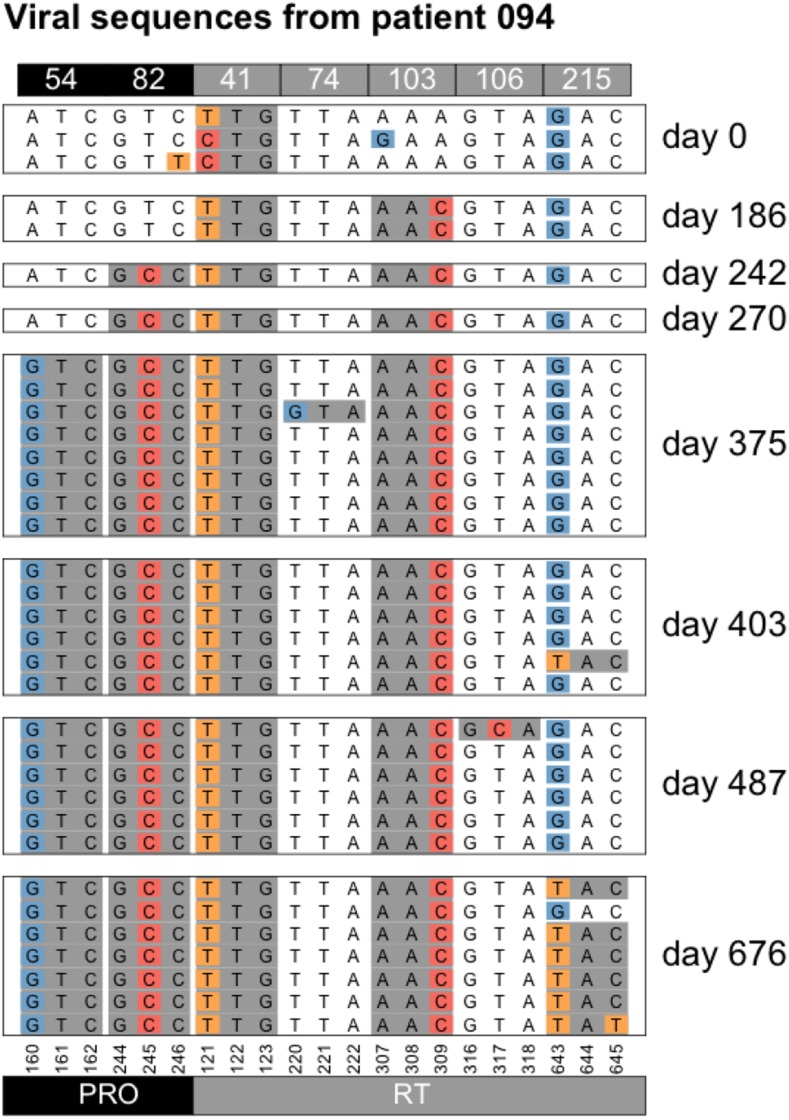
Accumulation of resistance mutations in patient 94.

We see several examples of several sweeps happening one after the other, leading to an accumulation of resistance mutations in the genome, a pattern that is sometimes called periodic selection (Atwood *et al.* 1951). For example, in patient 94, the day 0 sample shows resistance only at position 41, but in the two years that this patient was followed four new mutations are acquired by the virus (Table 1 and Figure 11). All sweeps are seen as complete sweeps, except the resistance mutation at codon 215 which is seen in 6 out of 7 sequences at the last time point (day 676).

**Table 1:** Accumulation of resistance mutations in patient 94

Patient 94	
Day	DRMs
0	41
186	41, 103
242	41, 103, 82
270	41, 103, 82
375	41, 103, 82, 54
403	41, 103, 82, 54
487	41, 103, 82, 54
676	41, 103, 82, 54, 215

### Clonal interference

Clonal interference is the effect when multiple beneficial mutations occur in a population on different genetic backgrounds. If recombination is rare or absent, then these mutations will at some point start to compete with each other, and ultimately, if one fixes in the population, the other one will be lost. When we see beneficial mutations (such as known resistance mutations) first increase in frequency, and then decrease drastically, this strongly suggests that clonal interference is causing the decrease in frequency. In the HIV populations we study here the rate of input of new mutations in the population must be fairly high (because soft sweeps from recurrent mutation happen) and recombination is quite rare (because simultaneous sweeps happen and because sweeps reduce diversity). It is therefore likely that clonal interference also happens in these adapting HIV populations. We start by showing an example of what our data would be expected to look like when clonal interference occurs (Figure 13). We show the alignment as before, and add a Muller plot (Muller 1932) or evolvogram (Angelova *et al.* 2018).

### Patient 84, Y188L interferes with K103N

We have shown the first few timepoints for patient 84 previously (Figure 14). Here we show a Muller diagram (Figure 14) and the sequences for the later time points (Figure 14). Mutations Y188L and K103N both lead to drug resistance against NNRTI drugs. Mutation Y188L requires two transversion mutations. K103N requires a single transversion mutation. In this patient, the data suggest that K103N occurred first, but the haplotype with Y188L had higher fitness and ultimately fixed. One sequence is observed with both K103N and Y188L. In Figure 14 M184V is also shown, which requires a single transition (A→G) and thus occurs *de novo* much more often as it has a higher mutation rate. M184V is fixed on both the K103N background and the Y188L background.

**Figure 12:**
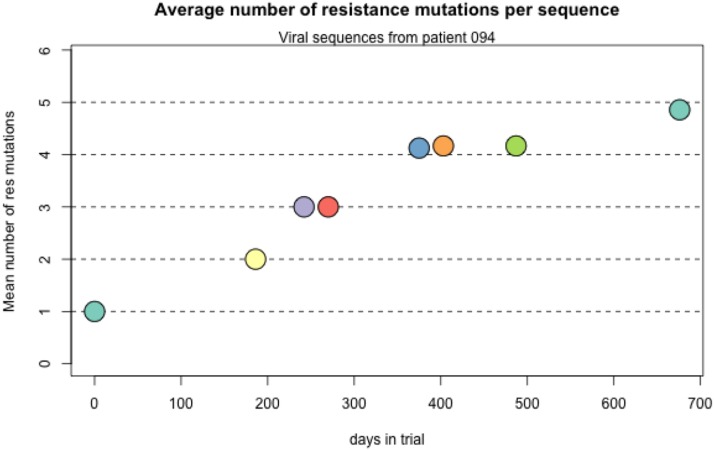
Accumulation of resistance mutations in patient 94. We show two other examples (patient 21 and patient 77) in the Additional Examples Supplement (figures 10 and 11).

**Figure 13:**
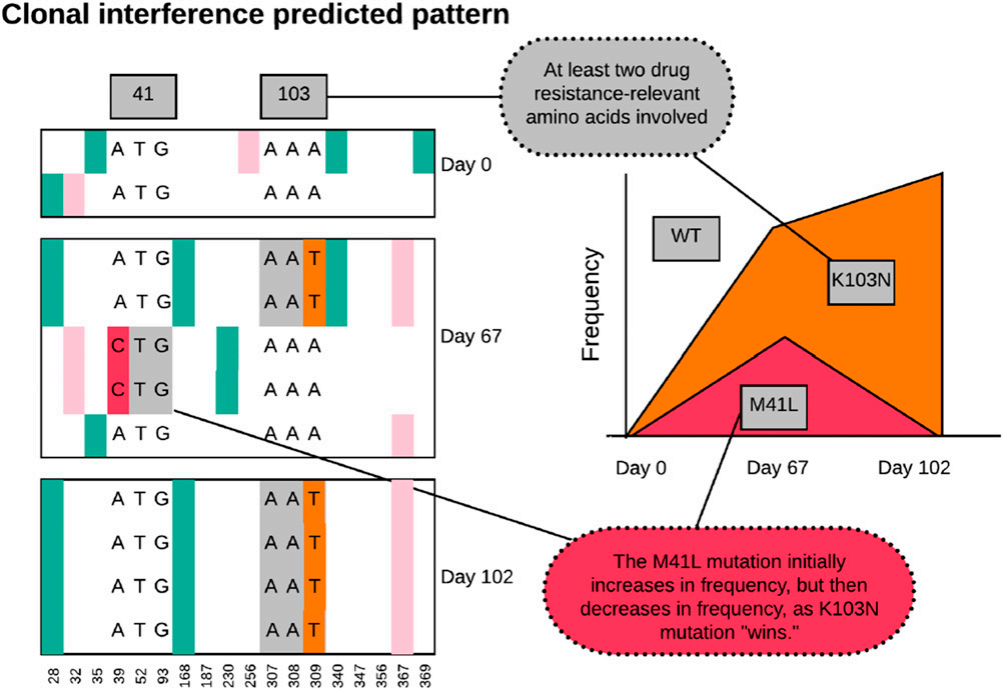
Alignment and Muller plot showing predicted pattern under clonal interference.

**Figure 14:**
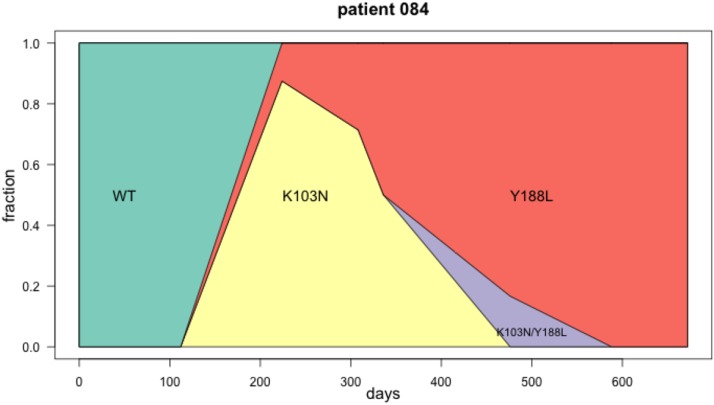
Muller plot showing clonal interference in patient 84. The K103N mutation first increases in frequency, while at the same time Y188L also appears in the population at low frequency (day 224). At the next time point (day 308) K103N has a slighly lower frequency, and Y188L a slightly higher frequency. This continues until at day 588, K103N is lost and Y188L is fixed. At one time point (day 476), we see one sequence with both, K103N and Y188L. It is unclear why this genotype doesn’t increase in frequency.

**Figure 15:**
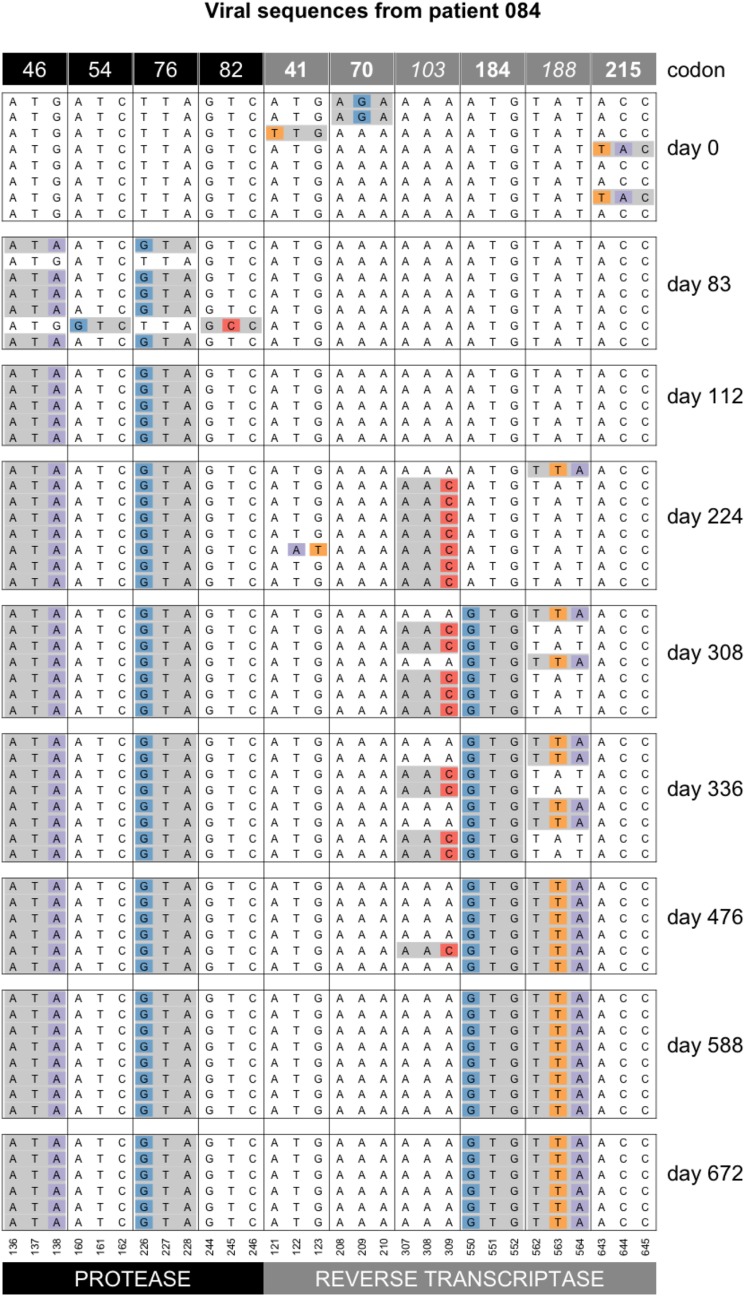
Sequence evolution in patient 84. At day 83, we see resistance mutations at positions 52 and 82 in protease. These mutations are then no longer observed at later time points. On days 224, 308, 336 and 476, we see mutation K103N in RT, it is in complete linkage disequilibrium with Y188L. On day 588, Y188L has fixed and K103 is no longer observed. Note that only drug-resistance related codons are shown in this figure. At one time point (day 476), we see one sequence with both, K103N and Y188L. It is unclear why this genotype doesn’t increase in frequency.

We show several other examples in the Additional Examples Supplement (patient 26, patient 72, patient 77, patient 89, patient 132 in Figures 12–16).

## Discussion

The goal of this paper is to provide examples of evolutionary dynamics of HIV within patients who are treated with antiretrovirals. We hope that the figures in this paper will be used in evolution and population genetics classes.

The data we used came from a dataset collected by Lee Bacheler and colleagues in the late 1990s. The patients were enrolled in several trials focused on the (then new) drug Efavirenz. The dataset contains sequences from 170 patients, but we focused on 118 patients with at least two sampling dates, and at least 5 sequences in total. We provide a supplement with figures showing all polymorphic nucleotides for all 118 patients. A caveat is that the treatment history of the patients is not always known. We have reason to believe that the annotation in the Genbank entries for the sequences is not always correct. Still, we included the treatment annotation in the supplemental figures.

In conclusion, we show a wide variety of patterns, specifically: soft sweeps, hard sweeps, softening sweeps and hardening sweeps, simultaneous sweeps, accumulation of mutations and clonal interference.
